# *Staphylococcus aureus* Coordinates Leukocidin Expression and Pathogenesis by Sensing Metabolic Fluxes via RpiRc

**DOI:** 10.1128/mBio.00818-16

**Published:** 2016-06-21

**Authors:** Divya Balasubramanian, Elizabeth A. Ohneck, Jessica Chapman, Andy Weiss, Min Kyung Kim, Tamara Reyes-Robles, Judy Zhong, Lindsey N. Shaw, Desmond S. Lun, Beatrix Ueberheide, Bo Shopsin, Victor J. Torres

**Affiliations:** a Department of Microbiology, New York University School of Medicine, New York, New York, USA; b Proteomics Resource Center, Office of Collaborative Science, New York University School of Medicine, New York, New York, USA; c Department of Cell Biology, Microbiology and Molecular Biology, University of South Florida, Tampa, Florida, USA; d Center for Computational and Integrative Biology and Department of Computer Science, Rutgers University, Camden, New Jersey, USA; e Department of Population Health, Division of Biostatistics, New York University School of Medicine, New York, New York, USA; f Department of Biochemistry and Molecular Pharmacology, New York University School of Medicine, New York, New York, USA; g Division of Infectious Diseases, Department of Medicine, New York University School of Medicine, New York, New York, USA

## Abstract

*Staphylococcus aureus* is a formidable human pathogen that uses secreted cytolytic factors to injure immune cells and promote infection of its host. Of these proteins, the bicomponent family of pore-forming leukocidins play critical roles in *S. aureus* pathogenesis. The regulatory mechanisms governing the expression of these toxins are incompletely defined. In this work, we performed a screen to identify transcriptional regulators involved in leukocidin expression in *S. aureus* strain USA300. We discovered that a metabolic sensor-regulator, RpiRc, is a potent and selective repressor of two leukocidins, LukED and LukSF-PV. Whole-genome transcriptomics, *S. aureus* exoprotein proteomics, and metabolomic analyses revealed that RpiRc influences the expression and production of disparate virulence factors. Additionally, RpiRc altered metabolic fluxes in the trichloroacetic acid cycle, glycolysis, and amino acid metabolism. Using mutational analyses, we confirmed and extended the observation that RpiRc signals through the accessory gene regulatory (Agr) quorum-sensing system in USA300. Specifically, RpiRc represses the *rnaIII* promoter, resulting in increased repressor of toxins (Rot) levels, which in turn negatively affect leukocidin expression. Inactivation of *rpiRc* phenocopied *rot* deletion and increased *S. aureus* killing of primary human polymorphonuclear leukocytes and the pathogenesis of bloodstream infection *in vivo.* Collectively, our results suggest that *S. aureus* senses metabolic shifts by RpiRc to differentially regulate the expression of leukocidins and to promote invasive disease.

## IMPORTANCE

The bicomponent pore-forming leukocidins play pivotal roles in the ability of *S. aureus* to kill multiple host immune cells, thus enabling this pathogen to have diverse tissue- and species-tropic effects. While the mechanisms of leukocidin-host receptor interactions have been studied in detail, the regulatory aspects of leukocidin expression are less well characterized. Moreover, the expression of the leukocidins is highly modular *in vitro*, suggesting the presence of regulators other than the known Agr, Rot, and *S. aureus* exoprotein pathways. Here, we describe how RpiRc, a metabolite-sensing transcription factor, mediates the repression of two specific leukocidin genes, *lukED* and *pvl*, which in turn has complex effects on the pathogenesis of *S. aureus*. Our findings highlight the intricacies of leukocidin regulation by *S. aureus* and demonstrate the involvement of factors beyond traditional virulence factor regulators.

## INTRODUCTION

*Staphylococcus aureus* is a daunting human pathogen that causes a range of diseases, from mild skin and soft tissue infections to debilitating and life-threatening bacteremia. In order to establish a successful infection, *S. aureus* secretes a variety of immunomodulatory proteins and virulence factors, a substantial number of which target leukocytes (1, 2). A complex family of these secreted proteins is the bicomponent pore-forming leukocidins (here referred to as leukocidins) (3, 4). These toxins consist of two different subunits that are secreted as water-soluble monomers. The binding subunit anchors to leukocytes through proteinaceous host receptors, recruits the other subunit, oligomerizes, and subsequently forms β-barrel pores within the host plasma membrane, leading to cell death (3, 4). Most *S. aureus* species carry five different leukocidins: leukocidin AB (LukAB, also known as LukHG), leukocidin ED (LukED), Panton-Valentine leukocidin (LukSF-PV, also known as PVL), and the gamma hemolysins HlgAB and HlgCB. Unique and central to the action of these leukocidins is their ability to lyse immune cells in a species-specific and cell-type-specific manner (3, 5). For example, while all of the leukocidins can target human polymorphonuclear leukocytes (hPMNs or neutrophils), LukAB, PVL, HlgAB, and HlgCB display greater tropism to human neutrophils than to murine neutrophils, whereas LukED is active against neutrophils from all of the species tested (6, 7). Similarly, the different leukocidins preferentially target specific immune cells on the basis of cell surface receptors (3, 5, 8–11).

Given their potential damaging effects, it is not surprising that the expression of leukocidins is tightly regulated by a highly complex, multifaceted regulatory network involving two-component systems (TCSs), winged helix-turn-helix (HTH) DNA-binding transcription factors, and at least one effector small RNA. Vital to the regulation of leukocidins is the master quorum-sensing regulatory system in *S. aureus*, which comprises the accessory gene regulator (Agr) proteins (12). At quorum (mimicked by postexponentially growing bacteria *in vitro*), *agr* produces an autoinducing peptide that, in the process of activating its own synthesis, leads to the production of the effector RNA molecule known as RNAIII (12, 13). RNAIII acts directly and indirectly on many virulence genes, up- or downregulating their translation and protein production (14, 15). Increased production of RNAIII leads to an increase in the abundance of exoproteins (including toxins and exoenzymes) and a decrease in the abundance of surface proteins involved in immune evasion (12–17). One critical target of RNAIII is the repressor of toxins (Rot) (14), which directly targets the leukocidin promoters (17, 18). In addition to the Agr-Rot regulatory network, the *S. aureus* exoprotein (Sae) expression TCS (SaeRS-TCS) has been implicated in the regulation of leukocidins (19–24). In contrast to Agr, which is a “self”-sensing system, the SaeRS-TCS responds to host-derived molecules and/or conditions likely encountered *in vivo*, such as pH, osmolarity, and neutrophil-derived proteins and peptides (21, 25, 26). Further linking Agr, Rot, and Sae is the observation that Rot negatively regulates one of the *saeRS* promoters, leading to robust repression of toxins (27). In sum, early in *S. aureus* infection, the *agr* locus is inactive, allowing enhanced production of surface molecules and adhesins, while toxins are repressed by Rot. At later stages of infection, the Agr system is activated and the expression of secreted virulence factors such as leukocidins is upregulated by RNAIII-mediated inhibition of Rot translation (7, 17). This circuit is then completed by relief of the inhibition of the *sae* locus, which leads to the further upregulation of leukocidins (23).

The success of *S. aureus* as a versatile pathogen relies in part on its ability to infect nearly all sites of the body. This adaptability depends on the ability of *S. aureus* to fine-tune the production of its virulence factors by sensing and responding to a diversity of external stimuli, leading to optimal pathogenesis, depending on the environment it is inhabiting (13, 28, 29). Therefore, identifying and characterizing regulators of toxins that sense and respond to disparate environmental conditions may shed light on *S. aureus* pathogenesis, enabling better therapeutic approaches for *S. aureus* clearance from specific infection sites*.*

In this study, we sought to identify transcriptional regulators of leukocidins in USA300, which is the leading cause of the current community-associated methicillin-resistant *S. aureus* epidemic in the United States (here referred to as USA300). We demonstrated that inactivation of a metabolic regulatory gene, *rpiRc*, increases *S. aureus* cytotoxicity for human neutrophils. RpiRc belongs to a family of transcriptional regulators with roles in the regulation of enzymes involved in sugar catabolism in many bacterial species. Using mutational analyses, we demonstrated that RpiRc differentially regulates the expression of leukocidins. Specifically, RpiRc is a potent repressor of the *lukED* and *lukSF-PV* loci. Importantly, we found that RpiRc-mediated gene regulation is critical to the fine-tuning of *S. aureus* pathogenesis.

## RESULTS

### The protein levels and promoter activities of the leukocidins vary during *S. aureus* growth *in vitro*.

**FIG 1:**
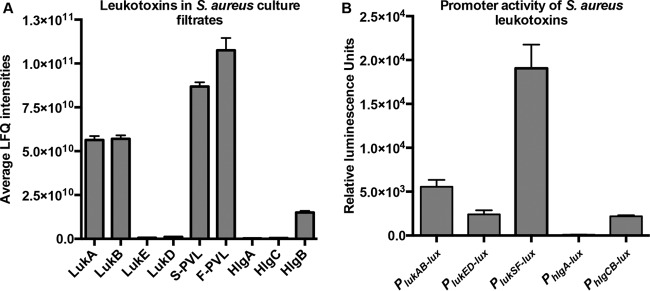
Leukocidin protein abundances and promoter activities vary during growth *in vitro.* (A) Quantitative mass spectrometry analyses (by LFQ) of postexponentially grown USA300 JE2 culture filtrates. (B) Leukocidin promoter activity in TSB measured by luminescence of postexponentially grown USA300 harboring plasmids of leukocidin promoter sequences fused to the luciferase gene. The values shown are averages of two independent experiments each performed with three colonies of each strain ± the standard deviation.

Postexponential-phase *S. aureus* cultures produce and secrete a variety of virulence factors, a major class of which are the leukocidins. Proteomic analyses of *S. aureus* culture filtrates revealed that the comparative levels of the five leukocidins differed greatly during the postexponential-phase growth of USA300 strain LAC in tryptic soy broth (TSB) (Fig. 1A). Specifically, LukAB and LukSF-PV were highly abundant in culture filtrates, while minimal levels of LukED and HlgACB were detected.

To test if these differential protein levels were due to differential regulation of the leukocidin promoters, we used toxin promoter sequences fused to luciferase reporter genes and measured the respective leukocidin promoter activity (17, 30). As shown in Fig. 1B, in the postexponential growth phase, the promoters of the different toxins were active to various degrees, and for the most part, expression profiles showed changes in protein levels (Fig. 1A). Among the leukocidins, the *lukSF* promoter activity and the corresponding PVL levels were the highest, whereas the *lukED* and *hlgA* promoters were minimally active, which was reflected in their respective protein amounts (Fig. 1A and B). Of note, the activation of the leukocidin promoters is highly dependent on the growth medium used (see Fig. S1 in the supplemental material). These results suggest that the differentially secreted leukocidins in USA300 are due primarily to variance among toxin promoter activities.

### Screen to identify transcriptional regulators that alter *S. aureus* cytotoxicity.

The differential activity of the leukocidin promoters may be due to the action of transcription factors at these promoters. In order to screen regulatory genes that may alter leukocidin production, we created a “regulator” sublibrary from the USA300 Nebraska transposon mutant library collection (31). This sublibrary consisted of 251 mutants of the JE2 strain (a laboratory version of USA300 LAC) that included gene products with any potential regulatory roles, including ones with nucleotide-binding domains, putative or confirmed HTH motifs, two-component regulatory systems, terminators, and antiterminators. For the functional annotations of the sublibrary, see Table S1 in the supplemental material.

All of the leukocidins are known to target hPMNs. Therefore, we screened supernatants collected from the 251 mutants for the ability to lyse hPMNs. Supernatants collected from the sublibrary grown for 3 h were used to intoxicate hPMNs isolated from four human donors. We found several mutants that exhibited altered cytotoxicity for hPMNs. For this study, we chose candidates that showed hypercytotoxicity with the goal of identifying novel repressors involved in the expression of *lukED* and *hlgACB* (see Table S1 in the supplemental material).

**FIG 2:**
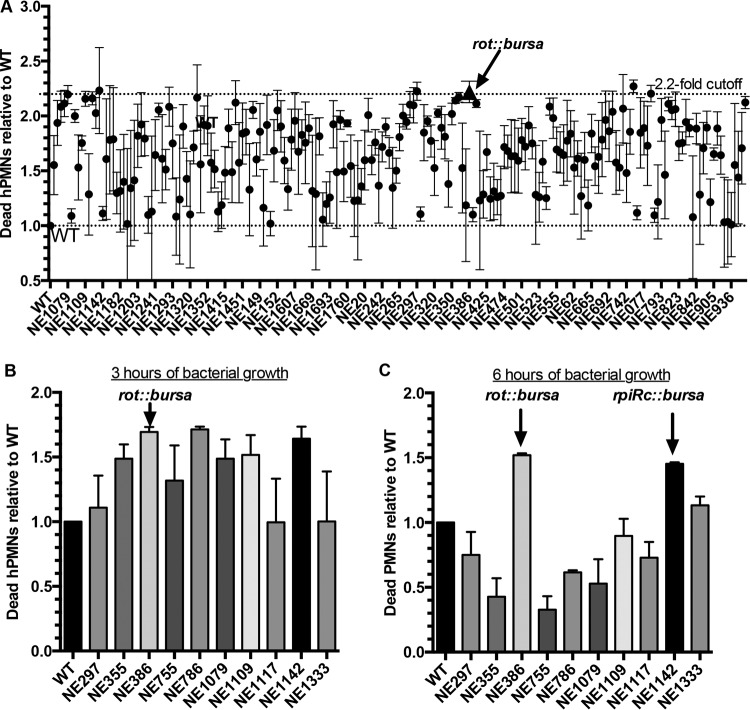
Identification of transcriptional regulators that enhance *S. aureus* cytotoxicity. (A) Primary intoxication screening of hPMNs with USA300 JE2 supernatants at a final concentration of 5% (vol/vol). Data points represent neutrophil death caused by an individual mutant relative to that caused by wild-type (WT) bacteria (lower dotted line). Supernatants from each mutant were tested on hPMNs from four donors, and cell viability was measured with CellTiter metabolic dye. A 2.2-fold cutoff was used to identify candidate mutants for further screening (upper dotted line). The data point indicated by the triangle represents the cytotoxicity of a *rot*::*bursa* mutant. (B and C) Validation intoxication screening of hPMNs isolated from two donors with supernatants from select *S. aureus* mutants. Wild-type and mutant bacteria were grown for 3 (B) and 6 (C) h postinoculation. Error bars indicate the standard error of the mean.

Compared to wild-type-induced cytotoxicity, mutants with changes in known leukocidin repressors, such as *rot* and *sigB*, were identified as hypercytotoxic in our screening (see Table S1 in the supplemental material), validating our assay. Of the mutations that caused increased cytotoxicity, 10 regulators led to neutrophil killing similar to that of a *rot*::*bursa* mutant (~2.2-fold increased cytotoxicity, Fig. 2A). In order to validate these data, we collected supernatants at both 3 and 6 h of bacterial growth. Of the 10 regulators tested, only *rot*::*bursa* and the mutation corresponding to NE1142 (*rpiRc*::*bursa*) caused increased cytotoxicity for hPMNs at both time points (Fig. 2B and C). Thus, we decided to focus on characterizing RpiRc and its repressive effects on *S. aureus* virulence.

### RpiRc controls the production of secreted virulence factors in *S. aureus*.

The RpiR class of proteins has been found in many different bacterial species, including Gram-negative *Escherichia coli* and *Pseudomonas putida* and Gram-positive *Bacillus subtilis* (32–34). This family of proteins is traditionally thought to include transcriptional regulators involved in sugar metabolism, although their regulons, binding sites, and exact functions are poorly characterized. The RpiR prototype contains an N-terminal HTH DNA-binding motif and a C-terminal sugar isomerase-sensing (SIS) domain (33, 35). A recent study identified three RpiR homologs in *S. aureus*, namely, *rpiRa*, *rpiRb*, and *rpiRc* (36). Mutations of these genes in methicillin-susceptible *S. aureus* strain UAMS-1 implicated these regulators in the control of the pentose phosphate pathway (PPP). Interestingly, inactivation of *rpiRc* also led to increased RNAIII synthesis and hemolysis and reduced biofilm formation (36).

**FIG 3:**
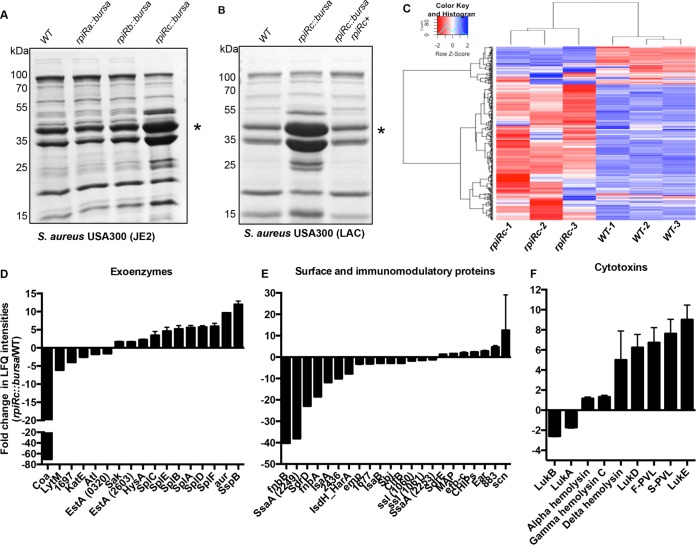
RpiRc is a potent regulator of *S. aureus* secreted proteins. (A) Exoprotein profiles of USA300 JE2 wild-type (WT) and *rpiR* mutant bacteria, as assessed by Coomassie staining. The asterisk indicates the approximate leukocidin protein size. (B) Exoprotein profile of USA300 LAC wild-type, *rpiRc*, and *rpiRc^+^* isogenic strains. (C) Heat map of LAC wild-type and *rpiRc* mutant secretomes as assessed by mass spectrometry. (D to F) Levels of exoenzymes (D), surface and immunomodulatory proteins (E), and cytotoxins (F) ± the standard deviation in exoproteomes of wild-type versus *rpiRc* mutant USA300 LAC.

To further evaluate the interactions among RNAIII, virulence, and RpiRc in USA300, we first examined the exoproteomes of wild-type and *rpiRc*::*bursa* mutant strains. We observed that various secreted proteins were differentially produced by the strains. The most dramatic difference in abundance was observed in protein bands corresponding to the size of leukocidins (~35 kDa). There were notably higher levels of proteins in that size range in the JE2 *rpiRc*::*bursa* culture filtrate than in that of wild-type JE2 (Fig. 3A), whereas mutations in the other *rpiR* genes had no effect on these toxins.

To demonstrate that the observed phenotype of the JE2 *rpiRc*::*bursa* mutant was due to the transposon-mediated disruption of *rpiRc*, the mutated allele was transduced into USA300 LAC strain AH1263 (referred to as LAC in this report), another erythromycin-sensitive LAC clone (37). Compared to the wild-type strain, the isogenic LAC *rpiRc*::*bursa* mutant also exhibited increased production of proteins that run at the size of leukocidins (Fig. 3B). Importantly, this phenotype was fully complemented by the insertion of *rpiRc* in single copy at the SaPI1 attachment site (referred to as the *rpiRc^+^* strain in this study) (Fig. 3B).

To gain better insight into the effects of RpiRc on exoprotein production in USA300, we analyzed the *in vitro* culture filtrates by mass spectrometry. Exoproteins were collected from three independent colonies of wild-type LAC and the *rpiRc* mutant strain grown to postexponential phase, and the protein profiles were analyzed by label-free quantitative mass spectrometry. We observed tremendous reproducibility among the biological replicates, as demonstrated by the clustering of the proteins in the heat map (Fig. 3C). We identified 96 (2-fold up or down in abundance) of the total 483 secreted proteins of USA300 to be altered in the mutant, of which 31 were positively impacted and 65 were negatively impacted in the *rpiRc* mutant (see Table S2 in the supplemental material). Among these proteins, we found increased production of most exoenzymes, such as proteases (4- to 6-fold increase), while a few exoenzymes, such as coagulase, were lower in abundance (Fig. 3D). Proteins involved in immune evasion and adhesion (including superantigens, Sbi, protein A, and ClfB) were, for the most part, lower in abundance in the *rpiRc* mutant (Fig. 3E). We found a stark increase in the production of cytotoxins (leukocidins and phenol-soluble modulins) in the *rpiRc* mutant (Fig. 3F). Interestingly, RpiRc seems to differentially regulate the production of leukocidins, as LukSF-PV and LukED were notably upregulated in the *rpiRc* mutant strain, whereas other leukocidins, such as gamma hemolysin and LukAB, were minimally impacted in this strain (Fig. 3F).

### Defining the RpiRc regulon.

**FIG 4:**
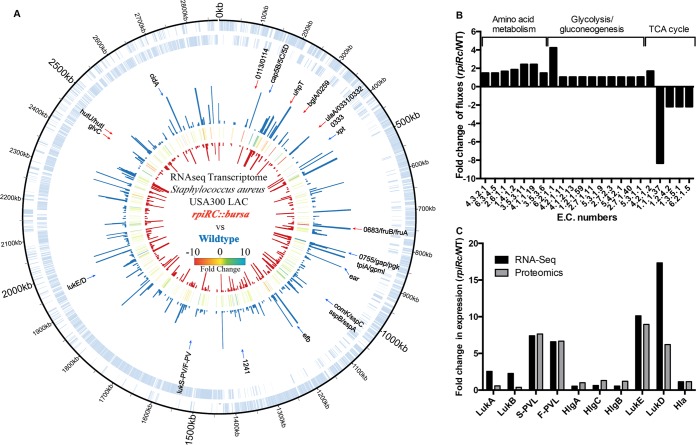
Defining the RpiRc regulon and the RpiRc-associated metabolic changes. RNA-Seq of USA300 wild-type and *rpiRc* mutant bacteria. (A) Genomic map depicting transcription profiles. The outer circle (blue) indicates the RPKM (number of reads per kilobase per million mapped reads) of the wild type, and the inner circle (red) indicates the RPKM of the *rpiRc* mutant. The center circle is a heat map showing the fold differences in expression. Genes that were 5-fold upregulated (blue arrows) or 5-fold downregulated (red arrows) are indicated. (B) Fold differences in metabolic flux between the wild type (WT) and the *rpiRc* mutant based on RNA-Seq results. The enzyme classification (E.C.) numbers represent specific enzymatic reactions and their corresponding pathways. (C) Fold differences in transcript (RNA-Seq) and protein (proteomics) abundance of the individual leukocidins between wild-type and *rpiRc* mutant bacteria.

The regulon of RpiRc in *S. aureus* is currently unknown. To gain a better understanding of the genes differentially regulated by RpiRc, we performed transcriptome sequencing (RNA-Seq) of RNA isolated from postexponential-phase cultures of wild-type and *rpiRc* mutant USA300. From the RNA-Seq analyses, we identified 476 genes altered (2-fold up- or downregulated) in the *rpiRc* mutant versus the wild-type strain. Among these genes, we found 82 to be repressed and 394 to be upregulated in the mutant (see Table S3 in the supplemental material). Genes that were 5-fold up- or downregulated in the *rpiRc*::*bursa* mutant are shown in Fig. 4A. Of these, the *lukED* and *lukSF-PV* leukocidin transcripts were drastically upregulated. In addition, genes involved in capsular polysaccharide biosynthesis and some proteases, such as *sspABC*, were also upregulated. In contrast, many genes involved in sugar metabolism and transport and surface virulence proteins (such as *spa*) were downregulated (see Table S3 in the supplemental material).

Since RpiRc is a regulator of metabolic enzymes, we wanted to determine if there were any general trends in the up- or downregulation of certain metabolic pathways. To address this question, we performed *in silico* metabolic profiling analyses of the transcriptomic data (38–40). As shown in Fig. 4B (see Table S4 in the supplemental material), we observed that three clusters of metabolic pathways were differentially activated in wild-type and *rpiRc* mutant bacteria. First, and notably, we observed a signature of decreased trichloroacetic acid (TCA) cycle activity in the *rpiRc* mutant. Second, and in contrast, several amino acid metabolic pathways were more active in the mutant than in the wild type. Third, we observed a slight but consistent activation of the glycolysis and gluconeogenesis pathways. While the fold differences in metabolic fluxes in glycolysis/gluconeogenesis between wild-type and *rpiRc* mutant bacteria are only moderate, we nevertheless observed that many genes in these two pathways were upregulated in the *rpiRc* mutant (Fig. 4B; see Table S4 in the supplemental material). Of note, we observed no growth defect *in vitro* when comparing the wild-type strain and the isogenic strain lacking *rpiRc* (see Fig. S2 in the supplemental material), consistent with observations reported previously (36). Taken together, mutation of *rpiRc* seems to lead to decreased activity of the TCA cycle and an increase in glycolysis/gluconeogenesis and certain amino acid biosynthetic pathways. In UAMS-1, RpiRc was observed to increase the expression and activity of some PPP genes (36). In our analyses, while there were no notable PPP shifts, we observed potential positive regulatory roles of RpiRc in the TCA cycle.

The RNA-Seq analyses also revealed differences in several virulence factors between the wild type and the mutant, the most dramatic of which was the differential expression of some leukocidins (Fig. 4A; see Table S3 in the supplemental material). Specifically, and consistent with the proteomic analyses, we observed a striking increase in the expression of *lukSF-PV* and *lukED* in the mutant (Fig. 4C), further validating that RpiRc is involved in the expression of these specific leukocidin-coding genes.

### RpiRc represses leukocidin expression by acting on Rot translation.

**FIG 5:**
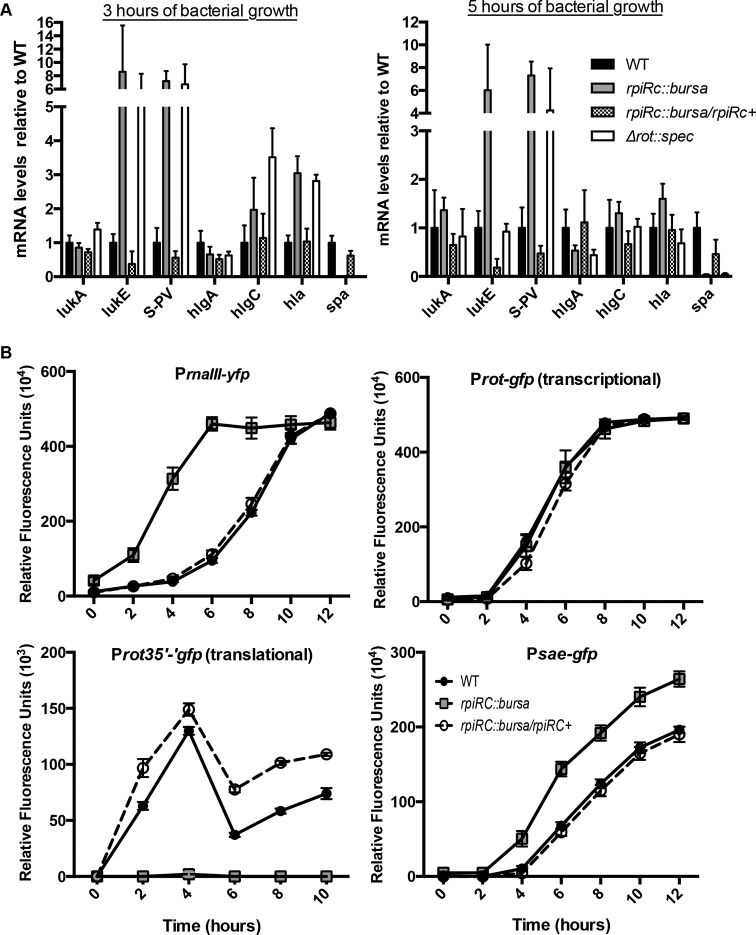
RpiRc regulates leukocidin expression by acting on Rot translation. (A) qRT-PCR analyses of transcripts in the USA300 LAC strains indicated. The relative abundance of the individual gene products was normalized to that of the wild type (WT). The experiment was performed with RNA extracted from three individual colonies, each assayed in triplicate, ± the standard deviation. (B) Time course comparing the expression of various promoters fused to luciferase or fluorescence genes. The values are averages of two independent experiments, each performed with three independent colonies of each strain, ± the standard deviation.

We next validated the RNA-Seq data on the leukocidins by quantitative real-time PCR (qRT-PCR). Consistent with the RNA-Seq and proteomic analysis results, levels of *lukS-PV* and *lukE* mRNAs were ~6- to 7-fold higher in the *rpiRc* mutant than in both the wild-type and *rpiRc^+^* strains (Fig. 5A)*.* In addition to the leukocidins, we also monitored the expression of alpha-toxin (encoded by *hla*), another important virulence factor in *S. aureus* (41). The *hla* gene was also found to be derepressed in the USA300 *rpiRc* mutant (Fig. 5A), data in line with the observation that deletion of *rpiRc* from strain UAMS-1 produces increased hemolytic activity (36).

To further dissect the signaling pathway(s) downstream of RpiRc, we measured the promoter activities of the master regulators of toxin gene expression, *rnaIII*, *sae*, and *rot*, in the *rpiRc* mutant. Consistent with the finding that RNAIII transcript levels are elevated in an *rpiRc* mutant of strain UAMS-1 (36), we observed 5-fold greater promoter activity of *rnaIII* in the USA300 *rpiRc* mutant than in the wild-type and *rpiRc^+^* strains (Fig. 5B). Moreover, we observed that while a *rot* transcriptional fusion was unaffected in the USA300 *rpiRc* mutant, a *rot* translational fusion was severely repressed in this background (Fig. 5B), consistent with the RNAIII-mediated translational regulation of *rot* (14, 42, 43). Lastly, consistent with decreased levels of Rot, we observed ~2-fold greater *sae* promoter activity in the *rpiRc* mutant than in the wild-type and *rpiRc^+^* strains (Fig. 5B).

We noticed that the transcriptional profiles of the leukocidins in the *rpiRc* mutant closely resembled that of the USA300 *rot* mutant (7, 17, 44), suggesting that RpiRc phenocopies the transcriptional effects of Rot (Fig. 5A). To further evaluate Rot functionality in the *rpiRc* mutant strain, we monitored the expression of a gene positively regulated by Rot, *spa*, which encodes the immune modulator protein A (45). Both RNA-Seq and qRT-PCR showed that *spa* levels were significantly lower in both the *rot* and *rpiRc* mutants (see Table S3 in the supplemental material; Fig. 5A). Collectively, these data suggest that in wild-type USA300, RpiRc represses the *agr* P3 promoter encoding RNAIII in a growth phase-dependent manner, which in turn leads to relief of the inhibition of *rot* translation and decreased *sae* expression, subsequently leading to the repression of specific leukocidins and other virulence factors.

### Mutation of *rpiRc* enhances *S. aureus* USA300-mediated virulence.

**FIG 6:**
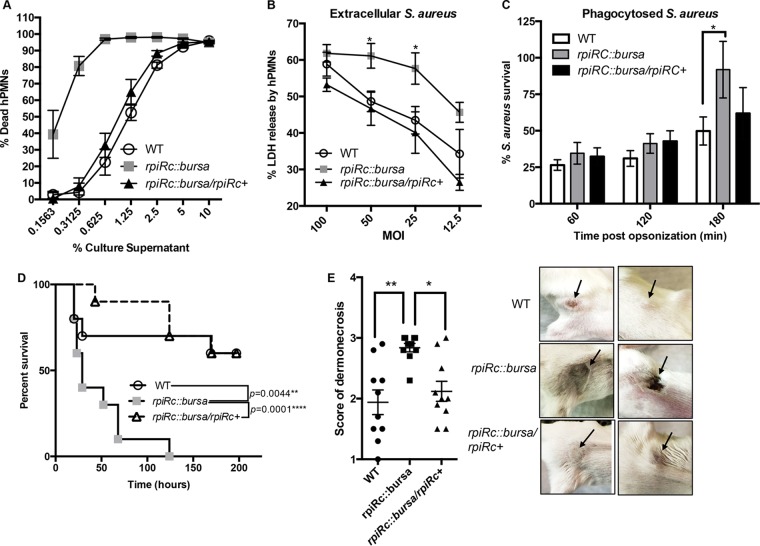
Inactivation of *rpiRc* enhances the virulence of USA300 LAC. (A) Intoxication of hPMNs (from six donors ± the standard of the mean) with a 2-fold titration of USA300 culture filtrates. Cell death was measured with CellTiter metabolic dye. (B) Infection of hPMNs (from three donors ± the standard of the mean) with the isogenic USA300 strains indicated under nonphagocytosing conditions. Cell death was measured as percent lactate dehydrogenase (LDH) release from lysed hPMNs. Statistical analyses were performed by ANOVA with Dunnett’s multiple comparison test. The asterisk indicates a *P* value of <0.05 for wild-type (WT) versus *rpiRc*::*bursa* bacteria and for *rpiRc*::*bursa* versus *rpiRc*::*bursa/rpiRc^+^* bacteria. (C) Growth rebound of the opsonized USA300 isogenic strains indicated during infection of hPMNs (from three donors ± the standard of the mean) at an MOI of 10 under phagocytosing conditions. Percent survival is the CFU count enumerated at the different time points relative to the start of infection. Statistical analyses were performed by ANOVA with Dunnett’s multiple comparison test. (D) Kaplan-Meier curve showing the percent survival of mice (10/group) infected retro-orbitally with ~5 × 10^7^ CFU of the isogenic strains indicated. Statistically significant differences between curves were determined by log rank (Mantel-Cox) test, and *P* values are shown. The results were corrected for multiple comparisons by using the Bonferroni-corrected threshold (assumed a statistical significance of 0.05 divided by two comparisons; 0.025). (E) Scores of murine skin lesions and dermonecrosis (five mice per group with two abscesses each) 48 h after intradermal infection with ~1 × 10^6^ CFU of the strains indicated. Each symbol is the average score per site of infection. Statistical analyses were performed with the Kruskal-Wallis test. *, *P* < 0.05; **, *P* < 0.005. Representative pictures of skin lesions at 48 h postinfection are shown on the right.

Next we elucidated the contribution of RpiRc to USA300-hPMN interactions, as neutrophils are innate immune cells critical for the containment of *S. aureus* (46). Consistent with the increased expression and production of secreted leukocidins described above, supernatants from the isogenic USA300 *rpiRc* mutant were more cytotoxic to hPMNs than were supernatants from wild-type USA300, a phenotype fully complemented in the *rpiRc^+^* strain (Fig. 6A).

To further explore the role of RpiRc during USA300-hPMN interactions, we infected hPMNs with wild-type and *rpiRc* mutant bacteria at different multiplicities of infection and monitored hPMN lysis. As shown in Fig. 6B, the *rpiRc* mutant also caused greater lysis of hPMNs than the wild-type and *rpiRc^+^* isogenic strains did. Lastly, we measured the survival of bacteria after inducing their phagocytosis by hPMNs. These studies revealed that the phagocytosed *rpiRc* mutant was better able to recover following ingestion by neutrophils than were the wild-type and *rpiRc^+^* strains (Fig. 6C). Thus, RpiRc in a wild-type scenario represses *S. aureus* virulence factors that enhance the killing of hPMNs.

We then tested the role of *rpiRc* in an *in vivo* murine bacteremia model. Mice were infected intravenously with the wild-type USA300, *rpiRc*::*bursa*, and *rpiRc*::*bursa/rpiRc^+^* strains, and their survival was monitored over time. Infection with the mutant strain led to significantly more mouse deaths than the wild-type and *rpiRc^+^* strains, strongly supporting our *ex vivo* data showing that RpiRc has critical roles in *S. aureus* pathogenesis (Fig. 6D).

To investigate whether RpiRc is important in other murine models, we also infected mice intradermally with the wild-type, *rpiRc*::*bursa*, and *rpiRc^+^* strains and monitored abscess formation and dermonecrosis postinfection. We observed that infection with the *rpiRc* mutant led to significantly higher dermonecrotic lesion scores than did infection with the wild-type and *rpiRc^+^* strains (Fig. 6E)*.* The increased virulence of the *rpiRc*::*bursa* strain in these models is likely to be due to the derepression of Hla (Fig. 3 to 5), as this toxin is critical for the pathogenesis of USA300 (41).

### Mutation of *rpiRc* enhances *S. aureus* virulence in a murine bloodstream infection model primarily through derepression of LukED.

**FIG 7:**
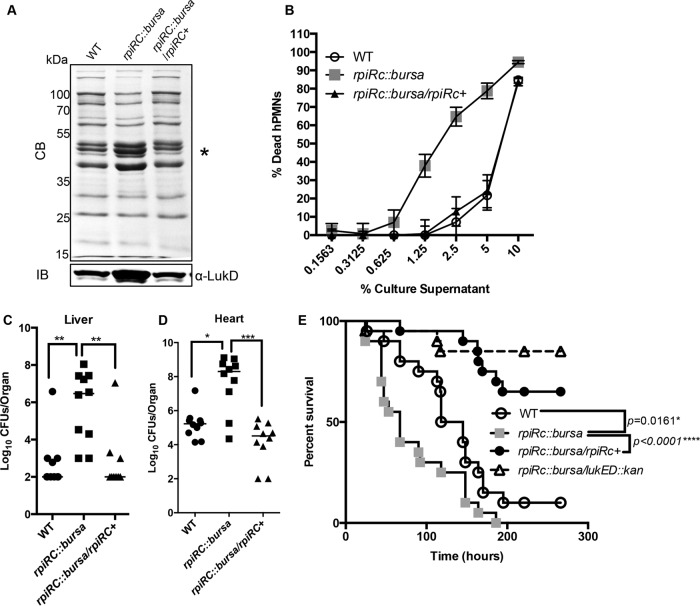
Mutation of *rpiRc* enhances *S. aureus* Newman virulence in a murine bloodstream infection model through derepression of LukED. (A) At the top is the exoprotein profile of postexponentially grown isogenic Newman strains stained with Coomassie blue (CB). The asterisk indicates the approximate leukocidin size. At the bottom is an immunoblot (IB) detecting LukD in the same samples. (B) Intoxication of hPMNs (from six donors ± the standard error of mean) with a 2-fold titration of Newman culture filtrates. Cell death was measured with CellTiter metabolic dye. (C and D) Bacteria recovered from the livers (C) and hearts (D) of female ND4 Swiss-Webster mice infected retro-orbitally with 1 × 10^7^ CFU of the strains indicated. Each symbol represents a mouse (*n* = 10). Analyses of statistical significance were performed with the Kruskal-Wallis test. *, *P* < 0.05; **, *P* < 0.005; ***, *P* < 0.0005. (E) Kaplan-Meier curve showing the percent survival of mice (20 per group) infected retro-orbitally with 2.5 × 10^7^ CFU of the isogenic strains indicated. Statistically significant differences between curves were determined by log rank (Mantel-Cox) test, and *P* values are shown. The results were corrected for multiple comparisons by using the Bonferroni-corrected threshold (assumed statistical significance of 0.05 divided by three comparisons; 0.0167), and the statistical significance of differences was determined. WT, wild type.

To understand the role of RpiRc in the virulence of non-USA300 strains, we created *rpiRc* mutant and complemented isogenic versions of *S. aureus* Newman, a strain that has been widely used to study the role of LukED in murine bacteremia models (6, 7, 9). Analyses of exoproteins produced by these isogenic strains revealed that, as in USA300, mutation of *rpiRc* in Newman results in greater production of leukocidins (Fig. 7A, top), including increased levels of LukD (Fig. 7A, bottom) than in the wild-type and complemented strains. Moreover, supernatants collected from *rpiRc* mutant Newman cultures were more cytotoxic to hPMNs than wild-type culture filtrates were (Fig. 7C).

Mice were infected systemically with sublethal doses of wild-type Newman, the *rpiRc* mutant, and the isogenic *rpiRc^+^* strain (1 × 10^7^ CFU), and bacterial burdens in organs were determined at 96 h postinfection. We observed significantly greater bacterial burdens in the hearts and livers of mice infected with the *rpiRc* mutant than in those of mice infected with the wild-type and complemented isogenic strains (Fig. 7C and D). Infection of mice with a higher dose (2.5 × 10^7^ CFU) of the *rpiRc* mutant led to more deaths of mice than did the infection of mice with the wild-type or complemented isogenic strain (Fig. 7E). The median survival of mice infected with the *rpiRc*::*bursa* mutant was 67 h, in contrast to the 131.5 h of wild-type-infected mice. The *rpiRc^+^* strain “overcomplemented” the lethality phenotype, potentially because of the slightly increased expression of *rpiRc* in this strain (see Fig. S3 in the supplemental material).

To directly elucidate the role of LukED in the observed increased virulence of the *rpiRc* mutant strain, we generated an *rpiRc lukED* double mutant and infected mice as described above. We noticed that, compared to the *rpiRc* mutant, the double mutant strain was severely attenuated (Fig. 7E), suggesting that the repression of *lukED* by RpiRc has important physiological consequences in bloodstream infection. Collectively, these data demonstrate that RpiRc is a critical transcriptional regulator involved in *S. aureus* pathogenesis.

## DISCUSSION

*S. aureus* secretes a diverse array of virulence factors (47), including the bicomponent pore-forming leukocidins (3) that target leukocytes. Studying the leukocidin mode of action, as well as the regulatory networks that govern their expression, will contribute to the understanding of *S. aureus* pathogenesis. Here, we undertook a screening strategy to identify potential regulators involved in the control of leukocidin expression (Fig. 2). We identified RpiRc, a metabolic transcriptional regulator, as a potent repressor of leukocidins (Fig. 3 to 5). Importantly, we demonstrated that RpiRc-mediated gene regulation impacts *S. aureus* pathogenesis in *in vitro*, *ex vivo*, and in *vivo* infection models. We observed that the transcriptional profiles of the *rpiRc* mutant were analogous to those of a *rot* mutant (44), with the upregulation of virulence factors such as exotoxins and downregulation of surface proteins like protein A.

In our preliminary screen to identify mutations that altered cytotoxicity, we set a cutoff of a 2.2-fold increase in cytotoxicity for selection of candidates. This was based on the cytotoxicity of the *rot* mutant, which resulted in a small fraction of mutants to be screened further (10 of the 251). It is evident that there are additional mutants identified by our screening that did not make the cutoff but are interesting regulators that will be the subject of future studies pursued in our laboratory.

The wild-type and *rpiRc* mutant strain exoprotein and transcriptomic profiles described here provide additional insight into the function of RpiRc in *S. aureus*. In the RNA-Seq analyses, in addition to the leukocidins, we observed >2-fold changes in genes involved in metabolism, sugar transport, translation, transcription, TCSs, and other regulators. While we did not validate all of the candidate genes, it is likely that RpiRc has direct or indirect effects on sugar metabolism, as it has been shown to be critical for the regulation of PPP genes (36). Validation and characterization of these targets may indicate other roles for RpiRc in *S. aureus* and may provide clues to links between metabolism and virulence.

The *S. aureus* virulence regulatory networks are complex and involve multifaceted environmental signals that dictate the fine-tuned expression of virulence factors, including leukocidins. The Agr-Rot pathway has been implicated as a critical component of leukocidin regulation (3, 7). Thus, it is not surprising that RpiRc also acts via this Agr-Rot pathway. However, there are many aspects of RpiRc-mediated regulation that have yet to be elucidated. First, the ligand(s) that triggers this regulatory pathway is unknown. A previous study of *S. aureus* RpiRc suggested that the ligand may be a metabolic intermediate, given its roles in metabolism and its N-terminal SIS domain (36). Our transcriptomic analyses indicate that there are drastic changes in genes involved in sugar uptake and metabolism in this mutant, further validating this idea. Second, it will be important to identify environmental conditions that trigger the activation or deactivation of RpiRc. Many transcriptional regulators that signal via the Agr system respond to a variety of conditions, including oxygen concentrations, amino acid availability, iron concentration status, and pH (29, 48, 49). Third, we have uncovered only one signaling pathway by which RpiRc acts on leukocidins. Whether this is the sole pathway for RpiRc-mediated regulation of virulence or if RpiRc can signal through other regulatory networks that operate under different *in vivo* conditions remains to be elucidated. Fourth, while it is clear that RpiRc represses RNAIII expression in *S. aureus* (Fig. 5B) (36), the molecular mechanism by which this occurs remains elusive. It is imperative to determine how RpiRc interacts with and regulates target promoters to determine direct versus indirect modes of gene regulation.

In order to be a successful pathogen, *S. aureus* has to adapt to the harsh environments encountered within the host. In recent years, interest in *S. aureus* metabolism has reemerged, as distinct links between metabolism and pathogenesis are increasingly identified (50, 51). In *S. aureus*, several transcriptional factors sense metabolites and regulate virulence in response to these signals. Examples of metabolite-sensing regulators in *S. aureus* are CcpA and CcpE*.* These carbon catabolite repressors sense glycolytic intermediates, and in addition to regulating uptake of nutrients such as glucose, they also regulate the synthesis of virulence factors (52, 53). Another well-studied nutrient sensor is the CodY transcriptional regulator, which responds to branched-chain amino acids and GTP in *S. aureus* (54). In response to nutrient availability, CodY regulates the synthesis of alpha-toxin and certain adhesins via the *agr* system (55). Importantly, inactivation of many of these regulators (including the ones cited above) alters *S. aureus* pathogenesis, supporting the notion that metabolism is intimately linked with the pathogenic lifestyle of this bacterium. The data presented here support the idea that RpiRc is a critical transcriptional regulator that may respond to various environmental conditions to increase, decrease, or fine-tune *S. aureus* virulence.

## MATERIALS AND METHODS

### Ethics statement.

Buffy coats were obtained from anonymous donors with informed consent from the New York Blood Center. Because all of the samples were collected anonymously prior to their delivery, the New York University Langone Medical Center (NYULMC) Institutional Review Board determined that our study was exempt from further ethics approval requirements. All animal experiments were reviewed and approved by the Institutional Animal Care and Use Committee of NYULMC. All experiments were performed according to NIH guidelines, the Animal Welfare Act, and U.S. federal law.

### Bacterial cultures and growth conditions.

*S. aureus* strains were routinely grown at 37°C on tryptic soy agar (TSA) or in TSB with antibiotic supplementation as specified. *E. coli* DH5α was used for cloning and propagation of plasmids. *E. coli* bacteria were grown in Luria-Bertani broth with appropriate antibiotics. Liquid cultures were grown in 5 ml of growth medium in 15-ml tubes incubated at a 45° angle with shaking at 180 rpm. For all experiments involving the growth of *S. aureus* bacteria, a 1:100 dilution of overnight cultures was subcultured into fresh medium.

### Construction of bacterial strains and plasmids.

For all of the strains, plasmids, and oligonucleotides used in this study, see Table S5 in the supplemental material. The LAC *rpiRc*::*bursa* mutant strain was generated by phage transduction of the JE2 *rpiRc*::*bursa* (NE1142) strain of the Nebraska Transposon Mutant Library with phage φ80 into wild-type, erythromycin-sensitive LAC clone AH1263 (37). Complementation of *rpiRc* on the chromosome was performed with suicide plasmid pJC1306 (kindly provided by John Chen), which is used to stably integrate DNA into the SaP1 site, resulting in a single-copy chromosomal insertion (56). The construction of these strains is described in Text S1 in the supplemental material. A LAC Δ*rot*::*spec* mutant strain was generated as described previously (17). The mutation was then transduced into erythromycin-sensitive LAC. The Newman *lukED*::*kan rpiRc*::*bursa* mutant strain was generated by transducing *lukED*::*kan* from strain Newman (as described in reference 7) with *rpiRc*::*bursa* from LAC (VJT42.71).

### Culture conditions for cytotoxicity screening.

The regulator mutant library was plated in a 96-well plate format on TSA. Overnight cultures of the mutants were grown in TSB on 2 independent days in 96-well round-bottom plates, subcultured into 96-well round-bottom plates, and grown for 3 h. The plates were centrifuged at 4,000 rpm, and the supernatants were collected into two different 96-well tissue culture-treated plates to give a 5% final concentration during intoxication and frozen. Supernatants from this regulator mutant library were then used to intoxicate hPMNs isolated from four different donors. In the validation screening, 10 mutants from the primary screening were grown as described above but for 3 and 6 h, and cytotoxicity to hPMNs from two donors was assayed.

### Cytotoxicity assay.

hPMNs were isolated as described in reference 57, and cytotoxicity assays were performed as described previously (17, 57). Briefly, 2 × 10^5^ hPMNs were added to a final volume of 100 µl/well of RPMI (Gibco) supplemented with 10 mM HEPES. Cells were intoxicated for 1 h at 37°C in 5% CO_2_. Ten microliters of CellTiter 96 Aqueous One Solution (CellTiter; Promega) was added, and the mixture was incubated at 37°C in 5% CO_2_ for 2 h. hPMN viability was assessed with a PerkinElmer EnVision 2103 Multilabel Reader.

### Exoprotein isolation, Coomassie staining, and immunoblotting.

The proteins in the culture supernatants of bacteria grown for 5 h were precipitated and analyzed as described in reference 17. Immunoblotting was performed with polyclonal antibodies against LukD (1:7,500), which were detected with a fluorescent Alexa Fluor 680-conjugated anti-rabbit antibody (1:25,000).

### Quantitative mass spectrometry analysis.

Wild-type and *rpiRc*::*bursa* mutant strain exoprotein isolates were analyzed in triplicate. Label-free quantification (LFQ) intensities obtained by mass spectrometry were log_2_ transformed, and all missing values were replaced with values from the normal distribution. *Z* scores were calculated for all values, hierarchical clustering was performed, and heat maps were generated. For detailed information, see Text S1 in the supplemental material.

### RNA isolation, RNA-Seq, and data analyses.

RNA isolation and sample preparation for RNA-Seq were performed as previously outlined by Carroll et al. (58). For detailed information on the RNA-Seq methods used, see Text S1 in the supplemental material.

### qRT-PCR.

A 10- to 100-ng sample of total RNA (depending on the abundance of the target gene) purified as described above was used to perform qRT-PCR in a one-step reaction with Reverse Transcriptase Mastermix (QuantiTect) and SYBR green master mix (Qiagen) in a 7300 Real-Time PCR system (Applied Biosystems). For the primers used to detect the specific mRNAs, see Table S5 in the supplemental material. Analysis was performed by the 2^−ΔΔ*CT*^ method, and target genes in each strain were normalized to the corresponding genes in wild-type cells.

### Reporter assays.

Strains containing reporter plasmids were grown overnight in different media containing 10 µg/ml chloramphenicol (to retain reporter plasmids) in 96-well round-bottom plates. Following subculture in fresh medium in 96-well black, flat-bottom plates (Corning), fluorescence and luminescence were measured with a PerkinElmer EnVision 2103 Multilabel Reader immediately after subculture (*T*_0_) and every 2 h for 10 to 12 h.

### Computational metabolic flux prediction.

A computational method called *E*-Flux2 was used to analyze the difference in intracellular metabolic fluxes between the wild-type and *rpiRc*::*bursa* mutant strains (for details, see Text S1 in the supplemental material). For a list of the metabolic pathways with significant changes and the complete list, see Table S4 in the supplemental material.

### *Ex vivo* assays of *S. aureus*-hPMN interactions.

Infection of hPMNs with extracellular *S. aureus* was performed as described previously (30), with RPMI supplemented with 10 mM HEPES and 5% human serum albumin at an hPMN concentration of 2 × 10^5^/well at 37°C in 5% CO_2_ for 1 h. The neutrophil-mediated *S. aureus* killing assay was performed as described previously (30). For details, see Text S1 in the supplemental material.

### Murine models of systemic and skin infections.

Five-week-old female ND4 Swiss-Webster mice (Harlan Laboratories) were anesthetized intraperitoneally with 250 to 300 µl of Avertin (2,2,2-tribromoethanol dissolved in *tert*-amyl alcohol and diluted to a final concentration of 2.5% [vol/vol] in sterile saline). For systemic and skin infections, 3-h *S. aureus* cultures were washed, resuspended in 1 × phosphate-buffered saline, and normalized for corresponding CFU counts. For systemic infections, 100 µl of inoculum was administered retro-orbitally. For experiments evaluating bacterial burdens, mice were euthanized with CO_2_ at 96 h postinfection and the organs indicated were harvested as described in reference 57. For acute/survival experiments, mice infected retro-orbitally were monitored every 4 to 6 h for signs of morbidity (hunched posture, lack of movement, paralysis, and inability to acquire food or water), at which time the animals were euthanized and survival curves were plotted over time (in hours). For skin infections, bacteria grown and processed as indicated above were mixed 1:1 with Cytodex 1 microcarrier beads (Sigma) in accordance with the manufacturer’s instructions and 100 µl was injected intradermally. Lesions were monitored every 12 h, pictures were taken, and 10 unbiased scientific volunteers were asked to score the lesions in a blind study. The scoring key was as follows: 1, no visible lesions; 2, milk skin lesions/dermonecrosis; 3, severe skin lesions/dermonecrosis.

### Statistical analyses.

The distribution of the data was first assessed to determine if it was normal. If the distribution was normal, then one-way or two-way analysis of variance (ANOVA; GraphPad Prism version 5.0; GraphPad Software) was used. Dunnett’s test was used for group comparisons as a follow-up to ANOVA. If the distribution of the data was nonparametric, we used the Kruskal-Wallis test to determine the statistical significance of the difference. The statistical significance of the difference between survival curves was determined by the Logrank test. The results were corrected for multiple comparisons by using the Bonferroni-corrected threshold (assumed a statistical significance of 0.05 divided by the number of comparisons).

## SUPPLEMENTAL MATERIAL

Figure S1  Promoter activities of leukocidins in various media. Leukocidin promoter activities in various media were measured by luminescence of postexponentially grown USA300 bacteria harboring plasmids of leukocidin promoter sequences fused to the luciferase gene. Values are averages of two independent experiments each performed with three colonies of each strain ± the standard deviation. Download Figure S1, PDF file, 0.05 MB

Figure S2  Growth curves of wild-type, *rpiRc*::*bursa*, and *rpiRc^+^* strains. Growth curves of overnight cultures of three independent colonies of the strains indicated at a 1:100 dilution in TSB medium were determined. Download Figure S2, PDF file, 0.03 MB

Figure S3  *rpiRc* mRNA levels in some of the strains used in this study. qRT-PCR analyses of *rpiRc* transcript levels in isogenic wild-type USA300, *rpiRc* mutant, *rpiRc^+^*, and *rot* mutant strains were performed. The relative abundances of the individual gene products were normalized to that of the wild type. The experiment was performed with RNA extracted from three individual colonies each assayed in triplicate. Download Figure S3, PDF file, 0.04 MB

Table S1  Regulator library screen and analyses. Table S1, XLSX file, 0.05 MB

Table S2  Proteomic analyses. Table S2, XLSX file, 0.1 MB

Table S3  RNA-Seq analyses. Table S3, XLSX file, 0.2 MB

Table S4  System-wide metabolic fluxes predicted by *E*-Flux2 in the wild-type and *rpiRc* mutant strains. Table S4, XLSX file, 0.1 MB

Table S5  Strains, oligonucleotides, and plasmids used in this study. Table S5, DOCX file, 0.03 MB

Text S1  Supplemental methods. Download Text S1, DOCX file, 0.03 MB
