# Supplementary figures and images for: Staphylococcus aureus Coordinates Leukocidin Expression and Pathogenesis by Sensing Metabolic Fluxes via RpiRc
Source: mBio. 2016 Jun 21;7(3):e00818-16. doi: 10.1128/mBio.00818-16 (PMC4916384; doi:10.1128/mBio.00818-16)

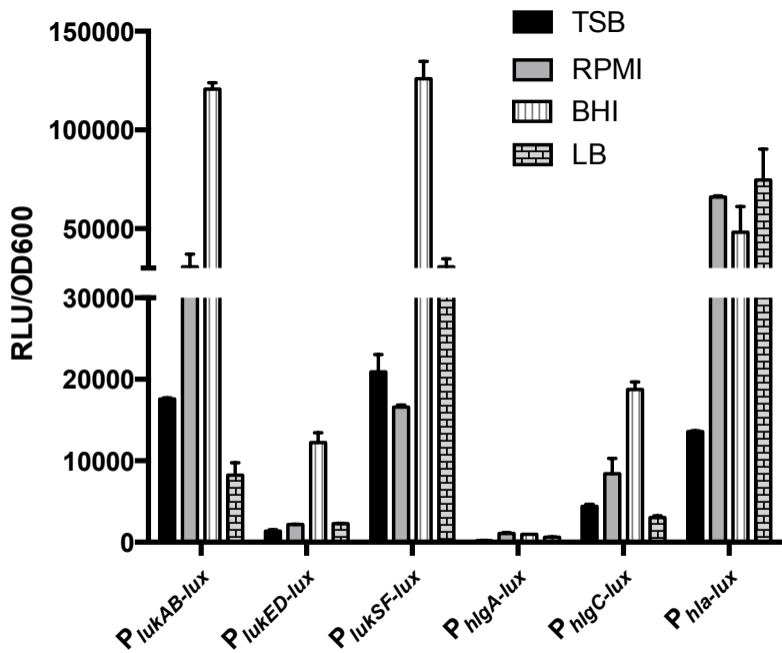

Supplement: Figure S1 — Promoter activities of leukocidins in various media. Leukocidin promoter activities in various media were measured by luminescence of postexponentially grown USA300 bacteria harboring plasmids of leukocidin promoter sequences fused to the luciferase gene. Values are averages of two independent experiments each performed with three colonies of each strain ± the standard deviation. Download [file mbo003162859sf1.pdf]

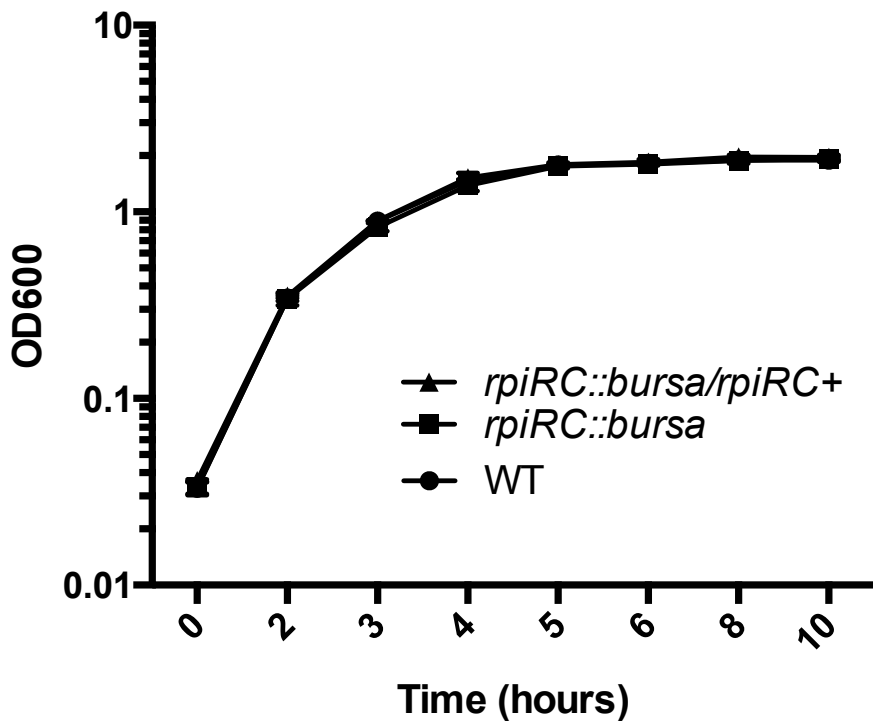

Supplement: Figure S2 — Growth curves of wild-type, rpiRc::bursa, and rpiRc+ strains. Growth curves of overnight cultures of three independent colonies of the strains indicated at a 1:100 dilution in TSB medium were determined. Download [file mbo003162859sf2.pdf]

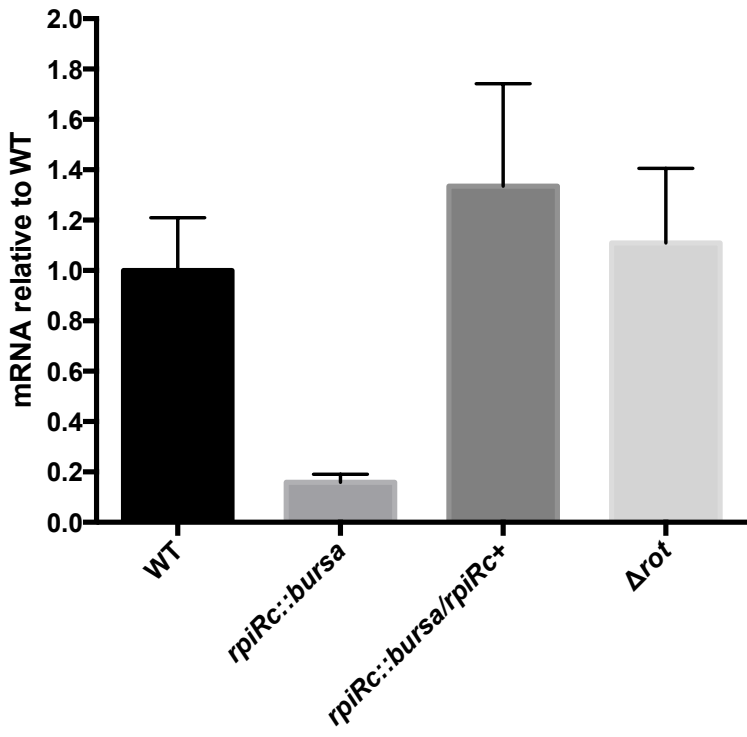

Supplement: Figure S3 — rpiRc mRNA levels in some of the strains used in this study. qRT-PCR analyses of rpiRc transcript levels in isogenic wild-type USA300, rpiRc mutant, rpiRc+, and rot mutant strains were performed. The relative abundances of the individual gene products were normalized to that of the wild type. The experiment was performed with RNA extracted from three individual colonies each assayed in triplicate. Download [file mbo003162859sf3.pdf]
