# Supplementary material for: Staphylococcus aureus Coordinates Leukocidin Expression and Pathogenesis by Sensing Metabolic Fluxes via RpiRc
Source: mBio. 2016 Jun 21;7(3):e00818-16. doi: 10.1128/mBio.00818-16 (PMC4916384; doi:10.1128/mBio.00818-16)
Supplement: Table S5 — Strains, oligonucleotides, and plasmids used in this study. [file mbo003162859st5.docx]

**Table S5.** **Strains, plasmids and oligonucleotides used in this study.**

**Strains**

| **VJT#** | **Strain Name** | **Description** | **Reference** |
| --- | --- | --- | --- |
| 15.77 | AH LAC | Erm^S^ USA300 parent strain | [1] |
| 31.81 | JE2 | Erm^S^ USA300 parent strain | [2] |
| 42.66 | JE2 *rpiRC::bursa* | JE2 carrying the *bursa aurealis* transposon in the *rpiRC* gene | [2] |
| 42.71 | AH LAC *rpiRC::bursa* | AH LAC carrying the *bursa aurealis* transposon in the *rpiRC* gene | This study |
| 45.21 | AH LAC pJC1306 | AH LAC carrying the pJC1306 vector in the SaPI1 site | This study |
| 45.22 | AH LAC *rpiRC::bursa* pJC1306 | AH LAC *rpiRC::bursa* carrying the pJC1306 vector in the SaPI1 site | This study |
| 45.23 | AH LAC *rpiRC::bursa* pJC1306/*rpiRC* | AH LAC *rpiRC::bursa* carrying pJC1306 expressing *rpiR*C in the SaPI1 site | This study |
| 45.24 | Newman pJC1306 | Newman carrying the pJC1306 vector in the SaPI1 site | This study |
| 45.25 | Newman *rpiRC::bursa* pJC1306 | Newman *rpiRC::bursa* carrying the pJC1306 vector in the SaPI1 site | This study |
| 45.26 | Newman *rpiRC::bursa* pJC1306/*rpiRC* | Newman *rpiRC::bursa* carrying pJC1306 expressing *rpiR*C in the SaPI1 site | This study |
| 17.37 | AH LAC  *rot::spec* | AH LAC containing spectinomycin insertion in *rot* | This study |
| 46.95 | Newman  *rpiRC::bursa*  *lukED::kan* | Newman *rpiRC::bursa* carrying pJC1306 expressing *rpiR*C in the SaPI1 site and *lukED::kanamycin* | This study |

**Plasmids**

| **Plasmid** | **Description** | **Reference** |
| --- | --- | --- |
| pXen/P*lukAB*-lux | Plasmid pXen1 with the *lukAB* promoter driving expression of the luciferase operon | [3] |
| pXen/P*lukSF*-lux | Plasmid pXen1 with the *lukSF-PVL* promoter driving expression of the luciferase operon | [3] |
| pXen/P*hlgCB*-lux | Plasmid pXen1 with the *hlgCB* promoter driving expression of the luciferase operon | [3] |
| pXen/P*hlgA*-lux | Plasmid pXen1 with the *hlgA* promoter driving expression of the luciferase operon | [3] |
| pXen/P*lukED*-lux | Plasmid pXen1 with the *lukED* promoter driving expression of the luciferase operon | [3] |
| pDB59 *agr*P3-*yfp* | pUC18-based plasmid with the *rnaIII* promoter driving expression of *yfp* | [4] |
| pOS1/P*sae*-s*gfp* | Plasmid pOS1 with the saeP1 promoter driving expression of super-folder *gfp* (*sgfp)* | [5] |
| pOS1/P*rot*-*sod*RBS-s*gfp* | Plasmid pOS1 with the *rot* promoter and the *sod* RBS driving expression of super-folder (*sgfp)* | [5] |
| pOS1/Prot-35aa-sgfp | Plasmid pOS1 with the *rot* promoter driving expression of the first 35 amino acids of Rot fused to s*gfp* |  |
| pJC1306 | Single-copy integration vector that inserts at the SaPI1 site | [6] |
| pJC1306/*rpiRC* | Plasmid pJC1306 expression rpiR from its native promoter | This study |

**Oligonucleotides**

| **VJT#** | **Description** | **Sequence (5’-3’)** |
| --- | --- | --- |
| 276 | *lukA* qRT-PCR | TTCCCAATATCATCCGGTGCTG |
| 277 | *lukA* qRT-PCR | GTTATCAGCAGCAACGACTCAAGC |
| 370 | *lukE* qRT-PCR | GAAATGGGGCGTTACTCAAA |
| 371 | *lukE* qRT-PCR | GAATGGCCAAATCATTCGTT |
| 286 | *hlgA* qRT-PCR | AATCGGAGGCAGTGGCTCATTCAA |
| 287 | *hlgA* qRT-PCR | GGACCAGTTGGGTCTTGTGCAAAT |
| 1311 | *S-PV* qRT-PCR | CCAATAAATTCTGGATTGAAGTTACCT |
| 1312 | *S-PV* qRT-PCR | GCTCAAGACAAAGCAACTTAAATGC |
| 290 | *spA* qRT-PCR | CAGCAAACCATGCAGATGCTA |
| 291 | *spA* qRT-PCR | GCTAATGATAATCCACCAAATACAGTTG |
| 1432 | *rpiRC* qRT-PCR | TAATCCCGTAGCACAGGCATC |
| 1433 | *rpiRC* qRT-PCR | TGTTGCTCCCATATGCATCTCAT |
| 278 | 16s rRNA qRT-PCR | TGAGATGTTGGGTTAAGTCCCGCA |
| 279 | 16s rRNA qRT-PCR | CGGTTTCGCTGCCCTTTGTATTGT |
| 1488 | *rpiRC* cloning into pJC1306 | AAAACTGCAGATCATCTAACGCATTA |
| 1490 | *rpiRC* cloning into pJC1306 | CGCGGATCCTTAATATTTGATAAATG |

**Literature Cited**

1. Boles, B.R., et al., *Identification of genes involved in polysaccharide-independent Staphylococcus aureus biofilm formation.* PLoS One, 2010. **5**(4): p. e10146.

2. Fey, P.D., et al., *A genetic resource for rapid and comprehensive phenotype screening of nonessential Staphylococcus aureus genes.* MBio, 2013. **4**(1): p. e00537-12.

3. DuMont, A.L., et al., *Staphylococcus aureus elaborates leukocidin AB to mediate escape from within human neutrophils.* Infect Immun, 2013. **81**(5): p. 1830-41.

4. Yarwood, J.M., et al., *Quorum sensing in Staphylococcus aureus biofilms.* J Bacteriol, 2004. **186**(6): p. 1838-50.

5. Benson, M.A., et al., *Rot and SaeRS cooperate to activate expression of the staphylococcal superantigen-like exoproteins.* J Bacteriol, 2012. **194**(16): p. 4355-65.

6. Chen, J., et al., *Single-copy vectors for integration at the SaPI1 attachment site for Staphylococcus aureus.* Plasmid, 2014. **76C**: p. 1-7.
