# Supplementary material for: Staphylococcus aureus Coordinates Leukocidin Expression and Pathogenesis by Sensing Metabolic Fluxes via RpiRc
Source: mBio. 2016 Jun 21;7(3):e00818-16. doi: 10.1128/mBio.00818-16 (PMC4916384; doi:10.1128/mBio.00818-16)
Supplement: Text S1 — Supplemental methods. Download [file mbo003162859s1.docx]

**Elaborated Supplemental methods**

Strain construction

All strains, plasmids and oligonucleotides are listed in Table S5. Fluorescent and luminescent reporter strains were generated by transformation of reporter plasmids into *S. aureus* via electroporation. Strain LAC *rpiRc::bursa* was generated by phage transduction of the JE2 *rpiRc::bursa* (NE1142) of the Nebraska Transposon Mutant Library using phage Φ80 into a wildtype, erythromycin-sensitive, LAC clone (AH1263, [1]). Complementation of *rpiRc* was performed using the suicide plasmid pJC1306 (kindly provided by Dr. John Chen), which is used to stably integrate DNA into the SaPI1 site, resulting in a single copy chromosomal insertion [2]. The *rpiRc* locus including its native promoter were amplified using primers 1488 and 1490, and cloned into pJC1306. To generate LAC/Newman and LAC/Newman *rpiRc::bursa* with pJC1306 or pJC1306/*rpiRc* inserted in the SaPI1 site, plasmids pJC1306 or pJC1306/*rpiRc* were transformed into RN4220 carrying pRN7023, which contains an integrase to incorporate the pJC1306 plasmid into the SaPI1 site [2]. The SaPI1 locus was transduced from the resulting strain into LAC/Newman and LAC/Newman *rpiRc::bursa* using phage Φ80 as described above.

RNA isolation, RNA-Seq and data analyses

Sample preparation for RNA sequencing was performed as previously outlined by Carroll *et al.* [3]. Briefly, RNA was isolated using the RNeasy kit (Qiagen) and DNA depleted employing the TURBO DNA-free kit (Ambion). The successful depletion of DNA was verified via PCR and the quality and concentration of the RNA evaluated using the Agilent 2100 Bioanalyzer and an RNA 6000 Nano Kit (Agilent). RNA from three biological replicates was pooled in equimolar amounts and ribosomal RNA removed by the successive application of the MICROBExpress (Ambion) and RiboZero (Epicentre) kits. The removal of rRNA was subsequently confirmed via the Agilent 2100 Bioanalyzer (RNA 6000 Nano Kit, Agilent). The rRNA-depleted RNA samples were then prepared for sequencing on the Ion Personal Genome Machine (PGM) System as described and employed previously [3, 4]. cDNA libraries were constructed with the Ion Total RNA-seq Kit v2 (Ion Torrent) and the libraries used to generate template-positive Ion Sphere Particles (ISPs) with the Ion PGM Template OT2 200 Kit (Ion Torrent). The template positive ISPs were loaded onto Ion 318 v2 chips (Ion Torrent) and sequencing runs performed with the Ion PGM Sequencing 200 Kit v2 (Ion Torrent). Data analysis was conducted using CLC Genomics Workbench (Qiagen) and the USA300-FPR757 reference genome (accession number: CP000255). RPKM values (Reads Per Kilobase per Million mapped reads) were generated for each gene, a quantile normalization approach applied [5] and a lower limit of 40 RPKM imposed. Genes that displayed fold-changes of ≥2 when comparing expression of the mutant to wildtype strain were included for further analysis.

Quantitative Mass spectrometry analysis

TCA precipitated protein pellets were reconstituted in 1X SDS-Laemmli buffer. Samples were reduced with 0.02 M dithiothreitol (pH 8) for 1hr at 57°C and subsequently alkylated with 0.05M iodoacetamide (pH 8) for 45 minutes in the dark at room temperature. For SDS clean up the Samples were loaded into a NuPAGE® 4-12% Bis-Tris Gel 1.0 mm (Life Technologies Corporation) and ran for approximately 5 minutes at 200 V. The resulting protein band was stained using GelCode Blue Stain Reagent (Thermo Scientific), and the band excised and cut into 1 x 1 mm pieces.

Protein gel bands were destained using 50% methanol in 100mM ammonium bicarbonate (pH = 8) (v/v). After at least 5 buffer changes of 15 minutes each, the gel pieces were dehydrated using acetonitrile and dried in a SpeedVac (Brand?). 300ng trypsin (Promega) were added and the gel pieces were covered with 100mM ammonium bicarbonate. The digestion was allowed to proceed overnight with gentle shaking at room temperature. The resulting peptide mixture was extracted from the gel pieces using a C18 Stage tip procedure, as previously described [6]. The desalted peptide mixture was concentrated in a SpeedVac concentrator and reconstituted in 0.5% acetic acid.

An aliquot of each sample was loaded onto a Acclaim PepMap 100 precolumn (75μm × 2cm, C18, 3μm, 100Å, Thermo Scientific) in-line with an EASY-Spray, PepMap column (75μm × 50cm, C18, 2μm, 100Å Thermo Scientific) with a 5μm emitter using the autosampler of an EASY-nLC 1000 (Thermo Scientific). Peptides were gradient eluted into a Q Exactive mass spectrometer (Thermo Scientific) using a 120min gradient from 2% solvent B to 40% solvent B. Solvent A was 2% acetonitrile in 0.5% acetic acid and solvent B was 90% acetonitrile in 0.5% acetic acid. MS1 spectra were acquired with a resolution of 70,000 (@m/z 200) an AGC target of 1e6, with a maximum ion time of 120ms, and scan range of 400 to 1,500 m/z. Following each MS1, 20 data-dependent high-resolution HCD MS2 spectra were acquired. All MS2 spectra were collected of precursors of charge states 2 – 5 using the following instrument parameters: resolution of 17,500 (@m/z 200), AGC target of 5e4, maximum ion time of 250ms, one microscan, 2 m/z isolation window, 30s dynamic exclusion list, and Normalized Collision Energy (NCE) of 27.

All acquired MS2 spectra were searched against a *S. aureus* USA300 UniProt database and proteins were quantified using the MaxQuant software suite [7, 8]. For the first search the peptide tolerance was set to 20 ppm and for the main search peptide tolerance was set to 4.5 ppm. Trypsin specific cleavage was selected with 2 missed cleavages. A PSM FDR of 1% and a Protein FDR of 1% was selected for identification. Label free quantitation (LFQ) was performed using a minimum ratio of 2 peptides per protein and limited tousing unique and razor peptides. Matching between runs was allowed with a 0.7 minute match window and a 20 minute alignment time window. Carbamidomethylation of Cys was added as a static modification. Oxidation of methionine and acetylation of the protein N-terminus were the allowed variable modifications.

Computational metabolic flux prediction

A computational method called E-Flux2 was used to analyze the difference in intracellular metabolic fluxes between the wildtype and the *rpiRc::bursa* mutant (described in [9-11] and elaborated in supplemental methods). Briefly, E-Flux2 infers a system-wide and condition-specific metabolic flux distribution by integrating transcriptomic data in a genome scale metabolic model [9]. It consists of two steps of optimization as follows:

|  | Step 1. E-Flux [10, 11] |  | Step 2. Minimization of *l*^2^ norm |  |
| --- | --- | --- | --- | --- |
|  | $z^{*}= max f'v$  $subject to\left\{ \begin{aligned} Sv=0 \\ a_{j}^{e}\leq v_{j}\leq b_{j}^{e} \end{aligned} \right.$ | **→** | $\min\sum_{j=1}^{n} {v_{j}}^{2}$  $subject to\left\{ \begin{aligned} Sv=0 \\ a_{j}^{e}\leq v_{j}\leq b_{j}^{e} \\ f'v = z^{*} \end{aligned} \right.$ |  |

In these calculations, $f$is a coefficient vector defining the organism’s objective function (in this study, biomass production was the objective function), $S$ is the stoichiometric matrix, $v$ is a flux vector representing the reaction rates of the $n$ reactions in the network, and $a_{j}^{e}$ and $b_{j}^{e}$are the minimum and maximum reaction rates through reaction $j$ that were set based on the expression level of the genes associated with $j$. After calculating the optimal biomass flux (denoted as $z^{*}$) in the first step, this method finds a unique metabolic flux distribution by minimizing the Euclidean norm of the flux vector in the second step.

Data obtained from transcriptomics analysis (described in detail above) of the two strains were used for E-Flux2 analysis. For the genome-scale metabolic model for *S. aureus* USA300 strain, we used *i*SB619 [12] with slight modifications in its gene-protein-reaction (GPR) associations. Specifically, since *i*SB619 was constructed based on the closely-related *S. aureus* N315 strain, genes in the model were converted to the corresponding orthologous USA300 genes. The predicted fluxes were normalized by growth rates of the two strains that were measured under the same conditions as the transcriptomic data. The list of metabolic pathways with significant changes is summarized and the full set can be found in Table S4.

Neutrophil-mediated killing of *S. aureus*

PBS-resuspended bacteria were opsonized in 20% normal human serum (Seracare) for 20 minutes at 37°C, after which they were centrifuged and re-suspended in RPMI supplemented with 10mM HEPES. Neutrophils were plated at 2.5x10^7^ cells/well in serum-coated tissue culture treated 96-well plates. Bacteria were then added to neutrophils and the samples centrifuged bring the bacteria in close proximity to the neutrophils for phagocytosis. Neutrophils were lysed with 1% saponin at indicated times post infection, serially diluted and plated on TSA (tryptic soy agar).

**References:**

1. Boles, B.R., et al., *Identification of genes involved in polysaccharide-independent Staphylococcus aureus biofilm formation.* PLoS One, 2010. **5**(4): p. e10146.

2. Chen, J., et al., *Single-copy vectors for integration at the SaPI1 attachment site for Staphylococcus aureus.* Plasmid, 2014. **76C**: p. 1-7.

3. Carroll, R.K., A. Weiss, and L.N. Shaw, *RNA-Sequencing of Staphylococcus aureus Messenger RNA.* Methods Mol Biol, 2015.

4. Weiss, A., et al., *The delta subunit of RNA polymerase guides promoter selectivity and virulence in Staphylococcus aureus.* Infect Immun, 2014.

5. McClure, R., et al., *Computational analysis of bacterial RNA-Seq data.* Nucleic Acids Res, 2013. **41**(14): p. e140.

6. Cotto-Rios, X.M., et al., *Deubiquitinases as a signaling target of oxidative stress.* Cell Rep, 2012. **2**(6): p. 1475-84.

7. Cox, J., et al., *Accurate proteome-wide label-free quantification by delayed normalization and maximal peptide ratio extraction, termed MaxLFQ.* Mol Cell Proteomics, 2014. **13**(9): p. 2513-26.

8. Luber, C.A., et al., *Quantitative proteomics reveals subset-specific viral recognition in dendritic cells.* Immunity, 2010. **32**(2): p. 279-89.

9. Kim, M.K., et al., *E-Flux2 and SPOT: Validated methods for inferring intracellular metabolic flux distributions from transcriptomic data.* BMC Systems Biology. *Submitted.* .

10. Colijn, C., et al., *Interpreting expression data with metabolic flux models: predicting Mycobacterium tuberculosis mycolic acid production.* PLoS Comput Biol, 2009. **5**(8): p. e1000489.

11. Brandes, A., et al., *Inferring carbon sources from gene expression profiles using metabolic flux models.* PLoS One, 2012. **7**(5): p. e36947.

12. Becker, S.A. and B.O. Palsson, *Genome-scale reconstruction of the metabolic network in Staphylococcus aureus N315: an initial draft to the two-dimensional annotation.* BMC Microbiol, 2005. **5**: p. 8.
